# Bis{μ-2-meth­oxy-6-[(methyl­imino)­meth­yl]phenolato}bis­({2-meth­oxy-6-[(methyl­imino)­meth­yl]phenolato}copper(II))

**DOI:** 10.1107/S1600536813025105

**Published:** 2013-09-21

**Authors:** Tetyana V. Sydoruk, Elena A. Buvaylo, Vladimir N. Kokozay, Olga Yu. Vassilyeva, Brian W. Skelton

**Affiliations:** a Department of Inorganic Chemistry, Taras Shevchenko National University of Kyiv, 64/13 Volodymyrska Street, Kyiv, Ukraine, 01601; b Centre for Microscopy, Characterisation and Analysis, University of Western Australia, 35 Stirling Highway, Crawley, WA 6009, Australia

## Abstract

The title compound, [Cu_2_(C_9_H_10_NO_2_)_4_], is built of discrete centrosymmetric dimers. The Cu^II^ atoms are each five coordinated by two deprotonated Schiff base ligands that are bonded differently to the metal atoms. Of the two phenolate O atoms, one is coordinated to one Cu^II^ atom, whereas another bridges the two metal atoms. The basal plane of the square pyramid around Cu^II^ atoms is formed by the imino N and phenolate O atoms of the bidentate and the monodentate/bidentate Schiff base ligands. The bridging phenolate oxygen occupies the apical position of the coordination sphere with a considerably longer Cu—O bond length. In the crystal, the dimeric mol­ecules pack relative to each other in such a way that the Cu_2_O_2_ planes of adjacent dimers are orthogonal.

## Related literature

For direct synthesis using metal powders and Schiff base ligands, see: Chygorin *et al.* (2012*a* ▶,*b* ▶) and references therein. For the structure of the Schiff base ligand 2-meth­oxy-6-imino­methyl­phenol, see: Chatziefthimiou *et al.* (2006 ▶). For structures of metal complexes of this Schiff base ligand, see: Meally *et al.* (2010 ▶, 2012 ▶); Zhang & Feng (2010 ▶).
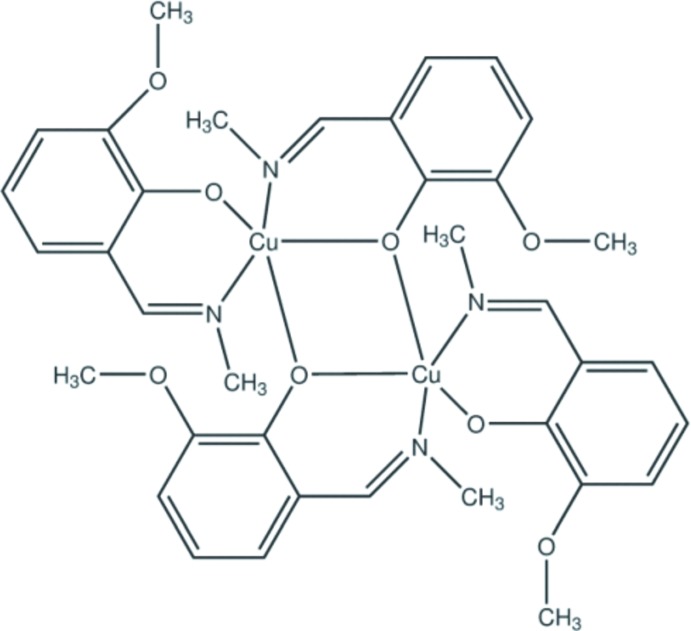


## Experimental

### 

#### Crystal data

[Cu_2_(C_9_H_10_NO_2_)_4_]
*M* *_r_* = 783.8
Orthorhombic, 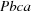

*a* = 10.1889 (12) Å
*b* = 15.2033 (5) Å
*c* = 21.6254 (9) Å
*V* = 3349.9 (4) Å^3^
*Z* = 4
Mo *K*α radiation
μ = 1.33 mm^−1^
*T* = 100 K
0.59 × 0.47 × 0.10 mm

#### Data collection

Oxford Diffraction Gemini diffractometer
Absorption correction: analytical [*CrysAlis PRO* (Agilent, 2011 ▶) based on Clark & Reid (1995 ▶)] *T*_min_ = 0.597, *T*_max_ = 0.88
110425 measured reflections
8673 independent reflections
7021 reflections with *I* > 2σ(*I*)
*R*_int_ = 0.045

#### Refinement

*R*[*F* ^2^ > 2σ(*F* ^2^)] = 0.031
*wR*(*F* ^2^) = 0.084
*S* = 1.05
8673 reflections
231 parameters
H-atom parameters constrained
Δρ_max_ = 0.64 e Å^−3^
Δρ_min_ = −0.37 e Å^−3^

### 

Data collection: *CrysAlis PRO* (Agilent, 2011 ▶); cell refinement: *CrysAlis PRO*; data reduction: *CrysAlis PRO*; program(s) used to solve structure: *SIR92* (Altomare *et al.*, 1994 ▶); program(s) used to refine structure: *SHELXL97* (Sheldrick, 2008 ▶); molecular graphics: *ORTEPII* (Johnson, 1976 ▶); software used to prepare material for publication: *WinGX* (Farrugia, 2012 ▶).

## Supplementary Material

Crystal structure: contains datablock(s) I, global. DOI: 10.1107/S1600536813025105/hg5344sup1.cif

Structure factors: contains datablock(s) I. DOI: 10.1107/S1600536813025105/hg5344Isup2.hkl

Additional supplementary materials:  crystallographic information; 3D view; checkCIF report

## Figures and Tables

**Table 1:** Selected bond lengths (Å)

Cu1—O21	1.9044 (7)
Cu1—O11	1.9243 (7)
Cu1—N27	1.9925 (8)
Cu1—N17	2.0032 (8)
Cu1—O11^i^	2.4329 (8)

Symmetry code: (i) 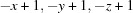.

**Fig. 1.**
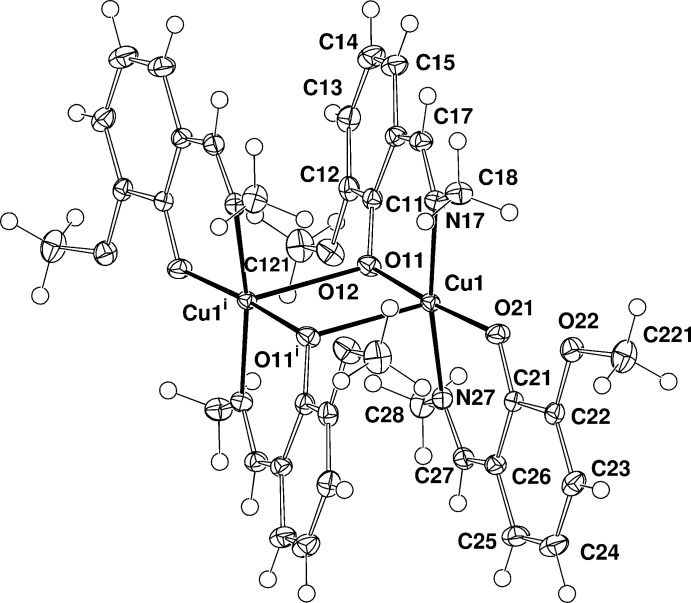
Molecular structure of the complex with the numbering scheme (the non-hydrogen atoms shown as 30% thermal ellipsoids). Symmetry code: (i) -x + 1, -y + 1, -z + 1.

**Fig. 2.**
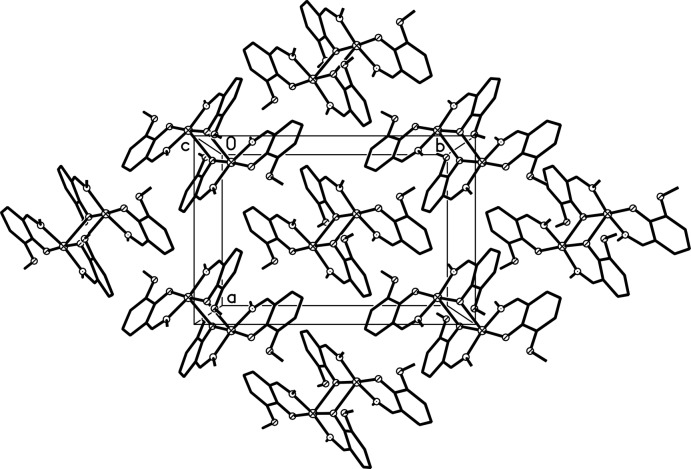
Packing diagram viewed down the c axis (CH and CH3 hydrogen atoms were omitted for clarity).

## supplementary crystallographic information

### 1. Comment

The Schiff base ligand 2-methoxy-6-iminomethylphenol (HL) (Chatziefthimiou *et al.* 2006) with various connectivity fashions is usually used as a multidentate linker between several metal centres thus affording electronic and magnetic exchanges. [Ni_7_], [Zn_7_] (Meally *et al.*, 2010), [Co_7_] (Meally *et al.*, 2012) and [Mn_7_] (Zhang *et al.*, 2010) complexes of HL (singly deprotonated at the phenolate site) with planar hexagonal disc-like cores which possess double-bowl metallocalix[6]arene topologies have shown to act as host cavities accommodating numerous guest solvent molecules.

The Schiff base, a bright yellow crystalline solid, is usually obtained by the standard method of condensation of the substituted salicylaldehyde with aqueous solution of methylamine in methanol (Meally *et al.*, 2010). In the present work, we used a mixture of 2-hydroxy-3-methoxy-benzaldehyde and methylamine hydrochloride to react with copper powder and transition metal salt, NiCl_2_^.^6H_2_O, in an attempt to prepare a heterometallic complex with HL ligand. Details of the used synthetic approach as well as its applications were given by Chygorin *et al.* (2012*a*) and Chygorin *et al.* (2012*b*). However, the monometallic [Cu_2_*L*_4_] 1 was isolated instead. As there is no evidence of the influence of NiCl_2_^.^6H_2_O on the formation of 1 it can be presumed that the given copper complex may be synthesized starting from metallic copper or copper salt as well. To the best of our knowledge no copper complexes of HL have been structurally characterized.

The molecular structure of 1 consists of discrete centrosymmetric dimers [Cu_2_*L*_4_] (Fig. 1). The copper atoms are five coordinated each by two deprotonated Schiff base ligands that are bonded differently to the metal centres. Of the two phenolate oxygen atoms, O21 is coordinated to one copper atom, whereas O11 bridges the two metal centres. The basal plane of the square pyramid around copper atoms is formed by the coordination of the imino nitrogen, N27, and phenolate oxygen, O21, atoms of the bidentate Schiff base ligand and N17 and O11 donor atoms of the tridentate *L* with Cu–O/N distances in the range 1.9044 (7)–2.0032 (8) Å (Table 1). The bridging phenolate oxygen O11{-*x* + 1,-*y* + 1,-*z* + 1} occupies the apical position of the coordination sphere with the bond distance of 2.4329 (8) Å. The elongation of the apical contact is typical for copper (II) complexes. The *trans* angles at the metal atom are equal to 169.53 (3) and 175.12 (3)°, the *cis* angles vary from 79.17 (3) to 105.57 (3)°. The deviation of the copper(II) ion from the basal plane is 0.13 Å. The bridge angle Cu1–O11–Cu1{-*x* + 1,-*y* + 1,-*z* + 1} involving the phenolate oxygen is 100.8 (2)°, the separation between the metal centres is about 3.37 Å.

In the crystal lattice, the dimeric molecules pack relative to each other in such a way that Cu_2_O_2_ planes of the adjacent dimers are orthogonal (Fig. 2).

### 2. Experimental

2-Hydroxy-3-methoxy-benzaldehyde (0.30 g, 2 mmol), CH_3_NH_2_^.^HCl (0.14 g, 2 mmol), NEt_3_ (0.3 ml, 2 mmol) were added to 20 ml of methanol and stirred magnetically for 30 min. After that copper powder (0.06 g, 1 mmol) and NiCl_2_^.^6H_2_O (0.23 g, 1 mmol) were added to the yellow solution and the mixture was heated to 323 K under stirring for an hour. The resulting green solution was filtered and allowed to stand at room temperature. Dark-green rhombic plates of the title compound were formed next day. They were collected by filter-suction, washed with dry Pr^i^OH and finally dried *in vacuo* (yield: 32%).

### 3. Refinement

Hydrogen atoms were placed at idealized positions (C–H = 0.95 Å, *U*_iso_H = 1.2*U*_eq_ C for CH, 0.98 Å, 1.5*U*_eq_ C for CH_3_) and refined as part of riding models.

### Crystal data

**Table:**

[Cu_2_(C_9_H_10_NO_2_)_4_]	*F*(000) = 1624
*M**_r_* = 783.8	*D*_x_ = 1.554 Mg m^−^^3^
Orthorhombic, *P**b**c**a*	Mo *K*α radiation, λ = 0.71073 Å
Hall symbol: -p 2ac 2ab	Cell parameters from 25414 reflections
*a* = 10.1889 (12) Å	θ = 3.7–37.4°
*b* = 15.2033 (5) Å	µ = 1.33 mm^−^^1^
*c* = 21.6254 (9) Å	*T* = 100 K
*V* = 3349.9 (4) Å^3^	Plate, dark green
*Z* = 4	0.59 × 0.47 × 0.10 mm

### Data collection

**Table:**

Oxford Diffraction Gemini diffractometer	8673 independent reflections
Graphite monochromator	7021 reflections with *I* > 2σ(*I*)
Detector resolution: 10.4738 pixels mm^-1^	*R*_int_ = 0.045
ω scans	θ_max_ = 37.5°, θ_min_ = 3.7°
Absorption correction: analytical [*CrysAlis PRO* (Agilent, 2011) based on Clark & Reid (1995)]	*h* = −17→17
*T*_min_ = 0.597, *T*_max_ = 0.88	*k* = −25→25
110425 measured reflections	*l* = −36→36

### Refinement

**Table:**

Refinement on *F*^2^	Primary atom site location: structure-invariant direct methods
Least-squares matrix: full	Secondary atom site location: difference Fourier map
*R*[*F*^2^ > 2σ(*F*^2^)] = 0.031	Hydrogen site location: inferred from neighbouring sites
*wR*(*F*^2^) = 0.084	H-atom parameters constrained
*S* = 1.05	*w* = 1/[σ^2^(*F*_o_^2^) + (0.0415*P*)^2^ + 0.9858*P*] where *P* = (*F*_o_^2^ + 2*F*_c_^2^)/3
8673 reflections	(Δ/σ)_max_ = 0.004
231 parameters	Δρ_max_ = 0.64 e Å^−^^3^
0 restraints	Δρ_min_ = −0.37 e Å^−^^3^

### Special details

**Table:**

Geometry. All s.u.'s (except the s.u. in the dihedral angle between two l.s. planes) are estimated using the full covariance matrix. The cell s.u.'s are taken into account individually in the estimation of s.u.'s in distances, angles and torsion angles; correlations between s.u.'s in cell parameters are only used when they are defined by crystal symmetry. An approximate (isotropic) treatment of cell s.u.'s is used for estimating s.u.'s involving l.s. planes.
Refinement. Refinement of *F*^2^ against ALL reflections. The weighted *R*-factor *wR* and goodness of fit *S* are based on *F*^2^, conventional *R*-factors *R* are based on *F*, with *F* set to zero for negative *F*^2^. The threshold expression of *F*^2^ > 2σ(*F*^2^) is used only for calculating *R*-factors(gt) *etc*. and is not relevant to the choice of reflections for refinement. *R*-factors based on *F*^2^ are statistically about twice as large as those based on *F*, and *R*- factors based on ALL data will be even larger.

### Fractional atomic coordinates and isotropic or equivalent isotropic displacement parameters (Å^2^)

**Table:**

	*x*	*y*	*z*	*U*_iso_*/*U*_eq_	
Cu1	0.402856 (11)	0.587478 (7)	0.514407 (5)	0.01235 (3)	
C11	0.32361 (9)	0.46134 (6)	0.42192 (4)	0.01446 (14)	
O11	0.42030 (7)	0.50521 (5)	0.44728 (3)	0.01630 (12)	
C12	0.33769 (10)	0.43410 (6)	0.35910 (4)	0.01654 (15)	
O12	0.45029 (8)	0.46267 (6)	0.33152 (3)	0.02223 (15)	
C121	0.47672 (13)	0.43092 (8)	0.27121 (5)	0.0262 (2)	
H12A	0.4814	0.3665	0.272	0.039*	
H12B	0.5606	0.4548	0.2567	0.039*	
H12C	0.4064	0.4494	0.2431	0.039*	
C13	0.24123 (11)	0.38471 (7)	0.33053 (5)	0.02007 (17)	
H13	0.2522	0.3668	0.2888	0.024*	
C14	0.12726 (12)	0.36098 (7)	0.36284 (5)	0.02237 (19)	
H14	0.0614	0.3269	0.343	0.027*	
C15	0.11093 (10)	0.38700 (7)	0.42308 (5)	0.01978 (17)	
H15	0.0337	0.3705	0.4448	0.024*	
C16	0.20753 (9)	0.43801 (6)	0.45312 (4)	0.01527 (15)	
C17	0.18368 (9)	0.46319 (6)	0.51653 (4)	0.01637 (15)	
H17	0.1114	0.4362	0.5367	0.02*	
N17	0.25120 (8)	0.51886 (5)	0.54829 (4)	0.01518 (13)	
C18	0.21142 (10)	0.53316 (7)	0.61269 (4)	0.01945 (17)	
H18A	0.1379	0.4942	0.6228	0.029*	
H18B	0.1843	0.5945	0.6181	0.029*	
H18C	0.2855	0.5204	0.6402	0.029*	
C21	0.44917 (9)	0.73470 (6)	0.59567 (4)	0.01441 (14)	
O21	0.37172 (7)	0.67187 (5)	0.57781 (4)	0.01810 (13)	
C22	0.41886 (9)	0.78000 (6)	0.65201 (4)	0.01576 (15)	
O22	0.30820 (7)	0.75006 (5)	0.68125 (3)	0.01921 (13)	
C221	0.25933 (13)	0.80269 (8)	0.73029 (5)	0.0256 (2)	
H22A	0.3214	0.8015	0.7649	0.038*	
H22B	0.1743	0.7796	0.744	0.038*	
H22C	0.2485	0.8634	0.7159	0.038*	
C23	0.49660 (11)	0.84784 (6)	0.67335 (5)	0.01998 (17)	
H23	0.4745	0.8767	0.7109	0.024*	
C24	0.60788 (11)	0.87448 (7)	0.64016 (5)	0.0236 (2)	
H24	0.6615	0.9209	0.6552	0.028*	
C25	0.63872 (11)	0.83310 (7)	0.58585 (5)	0.02131 (18)	
H25	0.7141	0.8513	0.5634	0.026*	
C26	0.56052 (9)	0.76367 (6)	0.56242 (4)	0.01585 (15)	
C27	0.58954 (9)	0.73111 (7)	0.50142 (5)	0.01682 (16)	
H27	0.6603	0.7582	0.4801	0.02*	
N27	0.52864 (8)	0.66860 (5)	0.47287 (4)	0.01607 (14)	
C28	0.56176 (12)	0.65605 (7)	0.40743 (5)	0.02199 (19)	
H28A	0.6356	0.6943	0.3964	0.033*	
H28B	0.4856	0.671	0.3818	0.033*	
H28C	0.5862	0.5945	0.4003	0.033*	

### Atomic displacement parameters (Å^2^)

**Table:**

	*U*^11^	*U*^22^	*U*^33^	*U*^12^	*U*^13^	*U*^23^
Cu1	0.01144 (5)	0.01349 (5)	0.01214 (5)	−0.00031 (3)	0.00112 (3)	−0.00053 (3)
C11	0.0148 (3)	0.0148 (3)	0.0138 (3)	0.0015 (3)	−0.0024 (3)	−0.0005 (3)
O11	0.0141 (3)	0.0204 (3)	0.0145 (3)	−0.0013 (2)	−0.0003 (2)	−0.0039 (2)
C12	0.0177 (4)	0.0175 (4)	0.0145 (4)	0.0035 (3)	−0.0022 (3)	−0.0015 (3)
O12	0.0198 (3)	0.0319 (4)	0.0149 (3)	0.0009 (3)	0.0021 (3)	−0.0060 (3)
C121	0.0323 (6)	0.0315 (5)	0.0147 (4)	0.0057 (4)	0.0027 (4)	−0.0042 (4)
C13	0.0247 (4)	0.0186 (4)	0.0169 (4)	0.0022 (3)	−0.0059 (3)	−0.0037 (3)
C14	0.0264 (5)	0.0189 (4)	0.0218 (4)	−0.0045 (4)	−0.0071 (4)	−0.0014 (3)
C15	0.0209 (4)	0.0183 (4)	0.0201 (4)	−0.0050 (3)	−0.0040 (3)	0.0008 (3)
C16	0.0156 (4)	0.0148 (3)	0.0153 (4)	−0.0013 (3)	−0.0022 (3)	0.0011 (3)
C17	0.0159 (4)	0.0165 (4)	0.0167 (4)	−0.0024 (3)	−0.0003 (3)	0.0023 (3)
N17	0.0148 (3)	0.0170 (3)	0.0137 (3)	−0.0011 (3)	0.0009 (2)	0.0004 (3)
C18	0.0201 (4)	0.0232 (4)	0.0151 (4)	−0.0040 (3)	0.0041 (3)	−0.0008 (3)
C21	0.0138 (3)	0.0128 (3)	0.0166 (4)	0.0005 (3)	−0.0003 (3)	0.0008 (3)
O21	0.0164 (3)	0.0173 (3)	0.0206 (3)	−0.0037 (2)	0.0050 (2)	−0.0050 (2)
C22	0.0177 (4)	0.0136 (3)	0.0160 (4)	0.0016 (3)	−0.0013 (3)	0.0010 (3)
O22	0.0204 (3)	0.0189 (3)	0.0183 (3)	0.0008 (3)	0.0036 (2)	−0.0030 (2)
C221	0.0350 (6)	0.0231 (5)	0.0187 (4)	0.0054 (4)	0.0062 (4)	−0.0026 (4)
C23	0.0256 (5)	0.0150 (4)	0.0193 (4)	−0.0008 (3)	−0.0057 (3)	−0.0003 (3)
C24	0.0269 (5)	0.0191 (4)	0.0248 (5)	−0.0074 (4)	−0.0070 (4)	0.0022 (4)
C25	0.0197 (4)	0.0198 (4)	0.0244 (5)	−0.0064 (3)	−0.0025 (3)	0.0046 (3)
C26	0.0145 (3)	0.0147 (3)	0.0184 (4)	−0.0011 (3)	−0.0010 (3)	0.0028 (3)
C27	0.0145 (4)	0.0166 (4)	0.0194 (4)	0.0007 (3)	0.0023 (3)	0.0042 (3)
N27	0.0162 (3)	0.0158 (3)	0.0162 (3)	0.0018 (3)	0.0034 (3)	0.0028 (3)
C28	0.0268 (5)	0.0220 (4)	0.0172 (4)	0.0019 (4)	0.0078 (4)	0.0030 (3)

### Geometric parameters (Å, º)

**Table:**

Cu1—O21	1.9044 (7)	C18—H18A	0.98
Cu1—O11	1.9243 (7)	C18—H18B	0.98
Cu1—N27	1.9925 (8)	C18—H18C	0.98
Cu1—N17	2.0032 (8)	C21—O21	1.2979 (11)
Cu1—O11^i^	2.4329 (8)	C21—C26	1.4135 (13)
C11—O11	1.3101 (11)	C21—C22	1.4333 (13)
C11—C16	1.4071 (13)	C22—O22	1.3705 (12)
C11—C12	1.4274 (13)	C22—C23	1.3799 (14)
O11—Cu1^i^	2.4329 (8)	O22—C221	1.4187 (13)
C12—O12	1.3640 (13)	C221—H22A	0.98
C12—C13	1.3825 (14)	C221—H22B	0.98
O12—C121	1.4165 (13)	C221—H22C	0.98
C121—H12A	0.98	C23—C24	1.4017 (16)
C121—H12B	0.98	C23—H23	0.95
C121—H12C	0.98	C24—C25	1.3689 (17)
C13—C14	1.4026 (16)	C24—H24	0.95
C13—H13	0.95	C25—C26	1.4163 (14)
C14—C15	1.3716 (15)	C25—H25	0.95
C14—H14	0.95	C26—C27	1.4396 (14)
C15—C16	1.4115 (14)	C27—N27	1.2922 (14)
C15—H15	0.95	C27—H27	0.95
C16—C17	1.4444 (14)	N27—C28	1.4673 (13)
C17—N17	1.2890 (12)	C28—H28A	0.98
C17—H17	0.95	C28—H28B	0.98
N17—C18	1.4667 (13)	C28—H28C	0.98
			
O21—Cu1—O11	175.12 (3)	N17—C18—H18A	109.5
O21—Cu1—N27	90.84 (3)	N17—C18—H18B	109.5
O11—Cu1—N27	90.16 (3)	H18A—C18—H18B	109.5
O21—Cu1—N17	87.65 (3)	N17—C18—H18C	109.5
O11—Cu1—N17	90.50 (3)	H18A—C18—H18C	109.5
N27—Cu1—N17	169.53 (3)	H18B—C18—H18C	109.5
O21—Cu1—O11^i^	105.57 (3)	O21—C21—C26	124.47 (9)
O11—Cu1—O11^i^	79.17 (3)	O21—C21—C22	118.40 (8)
N27—Cu1—O11^i^	92.04 (3)	C26—C21—C22	117.11 (8)
N17—Cu1—O11^i^	98.34 (3)	C21—O21—Cu1	127.51 (6)
O11—C11—C16	124.03 (8)	O22—C22—C23	124.49 (9)
O11—C11—C12	118.08 (9)	O22—C22—C21	114.20 (8)
C16—C11—C12	117.89 (8)	C23—C22—C21	121.32 (9)
C11—O11—Cu1	125.26 (6)	C22—O22—C221	116.50 (8)
C11—O11—Cu1^i^	113.86 (6)	O22—C221—H22A	109.5
Cu1—O11—Cu1^i^	100.84 (3)	O22—C221—H22B	109.5
O12—C12—C13	125.13 (9)	H22A—C221—H22B	109.5
O12—C12—C11	114.10 (8)	O22—C221—H22C	109.5
C13—C12—C11	120.76 (9)	H22A—C221—H22C	109.5
C12—O12—C121	117.00 (9)	H22B—C221—H22C	109.5
O12—C121—H12A	109.5	C22—C23—C24	120.60 (10)
O12—C121—H12B	109.5	C22—C23—H23	119.7
H12A—C121—H12B	109.5	C24—C23—H23	119.7
O12—C121—H12C	109.5	C25—C24—C23	119.49 (10)
H12A—C121—H12C	109.5	C25—C24—H24	120.3
H12B—C121—H12C	109.5	C23—C24—H24	120.3
C12—C13—C14	120.37 (9)	C24—C25—C26	121.35 (10)
C12—C13—H13	119.8	C24—C25—H25	119.3
C14—C13—H13	119.8	C26—C25—H25	119.3
C15—C14—C13	119.97 (10)	C21—C26—C25	120.12 (9)
C15—C14—H14	120	C21—C26—C27	121.59 (9)
C13—C14—H14	120	C25—C26—C27	117.94 (9)
C14—C15—C16	120.73 (10)	N27—C27—C26	126.30 (9)
C14—C15—H15	119.6	N27—C27—H27	116.8
C16—C15—H15	119.6	C26—C27—H27	116.8
C11—C16—C15	120.26 (9)	C27—N27—C28	116.49 (9)
C11—C16—C17	122.00 (8)	C27—N27—Cu1	123.28 (7)
C15—C16—C17	117.72 (9)	C28—N27—Cu1	120.15 (7)
N17—C17—C16	126.16 (9)	N27—C28—H28A	109.5
N17—C17—H17	116.9	N27—C28—H28B	109.5
C16—C17—H17	116.9	H28A—C28—H28B	109.5
C17—N17—C18	117.11 (8)	N27—C28—H28C	109.5
C17—N17—Cu1	123.97 (7)	H28A—C28—H28C	109.5
C18—N17—Cu1	118.90 (6)	H28B—C28—H28C	109.5

Symmetry code: (i) −*x*+1, −*y*+1, −*z*+1.

## References

[bb1] Agilent (2011). *CrysAlis PRO* Agilent Technologies, Yarnton, England.

[bb2] Altomare, A., Cascarano, G., Giacovazzo, C., Guagliardi, A., Burla, M. C., Polidori, G. & Camalli, M. (1994). *J. Appl. Cryst.* **27**, 435.

[bb3] Chatziefthimiou, S. D., Lazarou, Y. G., Hadjoudis, E., Dziembowska, T. & Mavridis, I. M. (2006). *J. Phys. Chem.* B**110**, 23701–23709. 10.1021/jp064110p 17125330

[bb4] Chygorin, E. N., Nesterova, O. V., Rusanova, J. A., Kokozay, V. N., Bon, V. V., Boča, R. & Ozarowski, A. (2012*a*). *Inorg. Chem.* **51**, 386–396. 10.1021/ic2017962 22129107

[bb5] Chygorin, E. N., Petrusenko, S. R., Kokozay, V. N., Omelchenko, I. V. & Shishkin, O. V. (2012*b*). *Acta Cryst.* E**68**, m233–m234. 10.1107/S1600536812003224 PMC3297208 22412398

[bb6] Clark, R. C. & Reid, J. S. (1995). *Acta Cryst.* A**51**, 887–897.

[bb7] Farrugia, L. J. (2012). *J. Appl. Cryst.* **45**, 849–854.

[bb8] Johnson, C. K. (1976). *ORTEPII* Report ORNL-5138. Oak Ridge National Laboratory, Tennessee, USA.

[bb9] Meally, S. T., McDonald, C., Karotsis, G., Papaefstathiou, G. S., Brechin, E. K., Dunne, P. W., McArdle, P., Power, N. P. & Jones, L. F. (2010). *Dalton Trans.* **39**, 4809–4816. 10.1039/b926704b 21491693

[bb10] Meally, S. T., McDonald, C., Kealy, P., Taylor, S. M., Brechin, E. K. & Jones, L. F. (2012). *Dalton Trans.* **41**, 5610–5616. 10.1039/c2dt12229d 22418687

[bb11] Sheldrick, G. M. (2008). *Acta Cryst.* A**64**, 112–122. 10.1107/S0108767307043930 18156677

[bb12] Zhang, S.-H. & Feng, C. (2010). *J. Mol. Struct.* **977**, 62–66.

